# Supplementary material for: Impulse control disorders and other non-motor symptoms in Sri Lankan patients with Parkinson’s disease
Source: PLoS One. 2024 Oct 18;19(10):e0312342. doi: 10.1371/journal.pone.0312342 (PMC11488724; doi:10.1371/journal.pone.0312342)
Supplement: S1 Table — (DOCX) [file pone.0312342.s002.docx]

Supplementary table 1. Variables that were collected in this study and their definitions.

| Variable | Definition |
| --- | --- |
| Age (Years) |  |
| Gender |  |
| Marital status |  |
| Education level |  |
| Monthly income |  |
| Employment status after Parkinson |  |
| Presence of financial dependents at home |  |
| Availability of a caregiver at home |  |
| Age of onset of Parkinson’s disease (Years) |  |
| Duration of Parkinson’s disease (Years) |  |
| Modified Hoehn and Yahr Staging |  |
| L-Dopa - Levodopa equivalent daily doses (LEDD) | Were calculated as described previous research (1, 2) |
| Levodopa duration (months) |  |
| Dopamine agonist - Levodopa equivalent daily doses (DA -LEDD) | Were calculated as described previous research (1, 2) |
| Dopamine agonist duration (Months) |  |
| Amantadine dose |  |
| Amantadine duration |  |
| Benzhexole dose |  |
| Benzhexole duration |  |
| Body Mass index |  |
| Current or history of smoking | Self-lifetime usage of at least 5 pack-years of cigarettes (3) |
| Family History of smoking | Parents, siblings, or children- lifetime usage of at least 5 pack-years of cigarettes (3) |
| Current/Past history of alcohol use disorder | Self- alcohol use disorder was assessed using DSM-V criteria (4). |
| Family history of alcohol use disorder | Parents, siblings, or children- alcohol use disorder was assessed using DSM-V criteria (4). |
| Current/Past history of substance abuse | Self- substance ( other than alcohol) use disorder was assessed using DSM-V criteria (4). |
| Family history of substance abuse | Parents, siblings, or children - substance ( other than alcohol) use disorder was assessed using DSM-V criteria (4). |
| History of psychiatric disorders | Past history of self – depression, psychosis of depression diagnosed using DSM-V criteria (4). |
| Family history of psychiatric illness | Past history of parents, siblings, or children - – depression, psychosis of depression diagnosed using DSM-V criteria (4). |

References

1. Rabinak CA, Nirenberg MJ. Dopamine agonist withdrawal syndrome in Parkinson disease. Arch Neurol. 2010;67(1):58-63.

2. Tomlinson CL, Stowe R, Patel S, Rick C, Gray R, Clarke CE. Systematic review of levodopa dose equivalency reporting in Parkinson's disease. Mov Disord. 2010;25(15):2649-53.

3. Bastiaens J, Dorfman BJ, Christos PJ, Nirenberg MJ. Prospective cohort study of impulse control disorders in Parkinson's disease. Mov Disord. 2013;28(3):327-33.

4. American Psychiatric A. Diagnostic and Statistical Manual of Mental Disorders2013.
