# Supplementary material for: Impulse control disorders and other non-motor symptoms in Sri Lankan patients with Parkinson’s disease
Source: PLoS One. 2024 Oct 18;19(10):e0312342. doi: 10.1371/journal.pone.0312342 (PMC11488724; doi:10.1371/journal.pone.0312342)
Supplement: S2 Table — (DOCX) [file pone.0312342.s003.docx]

Supplementary table 2 = Frequency of non-motor symptoms reported by patients.

| Symptom | Present | Percentage |
| --- | --- | --- |
| Fatigue/ lack of energy | 169/192 | 88% |
| Pain/ unpleasant sensation | 142/192 | 74% |
| Anxiety | 134/192 | 70% |
| Daytime sleepiness | 132/192 | 69% |
| Insomnia | 125/192 | 65% |
| Depression | 121/192 | 63% |
| Cognitive impairment | 112/192 | 58% |
| Altered bowel habits | 104/192 | 54% |
| Apathy | 102/192 | 53% |
| Eating / swallowing difficulty | 81/192 | 42% |
| Urinary symptoms | 80/192 | 42% |
| Drooling of saliva | 73/192 | 38% |
| Rapid eye movement disorder | 59/192 |  |
| Hyposmia | 48/192 | 25% |
| Hallucinations | 40/192 | 21% |
| Psychosis | 17/192 | 9% |
