# Supplementary material for: Impulse control disorders and other non-motor symptoms in Sri Lankan patients with Parkinson’s disease
Source: PLoS One. 2024 Oct 18;19(10):e0312342. doi: 10.1371/journal.pone.0312342 (PMC11488724; doi:10.1371/journal.pone.0312342)
Supplement: S3 Table — (DOCX) [file pone.0312342.s004.docx]

Supplementary table 3 = Self-perceived impact of each non-motor symptoms, scored on a 10-point Likert scale

| Symptom | Median | Interquartile range |
| --- | --- | --- |
| Fatigue/ lack of energy | 5 | 3-8 |
| Pain/ unpleasant sensation | 4 | 0-6 |
| Anxiety | 2 | 0-6 |
| Daytime sleepiness | 3 | 0-6 |
| Insomnia | 3 | 0-7 |
| Depression | 2 | 0-7 |
| Cognitive impairment | 3 | 0-6 |
| Altered bowel habits | 1 | 0-5 |
| Apathy | 1 | 0-5 |
| Eating / swallowing difficulty | 0 | 0-3 |
| Urinary symptoms | 0 | 0-2 |
| Drooling of saliva | 0 | 0-1 |
| Rapid eye movement disorder | 0 | 0-1 |
| Hyposmia | 0 | 0-1 |
| Hallucinations | 0 | 0-0 |
| Psychosis | 0 | 0-0 |
